# Subacute and subchronic oral toxicity assessments of *Acridocarpus smeathmannii* (DC.) Guill. & Perr. root in Wistar rats

**DOI:** 10.1016/j.toxrep.2019.01.005

**Published:** 2019-01-30

**Authors:** O.E. Kale, O. Awodele, A.J. Akindele

**Affiliations:** a Department of Pharmacology, Therapeutics & Toxicology, College of Medicine, University of Lagos, P.M.B, 12003, Idi-Araba, Lagos, Nigeria; b Department of Pharmacology, Benjamin S. Carson (Snr.) School of Medicine, Babcock University, Ilishan-Remo, Ogun State, PMB, Ikeja, 21244, Nigeria

**Keywords:** COX-2, cyclooxygenase-2, ELISA, enzyme-linked immunosorbent assay, FSH, Follicle stimulating hormone, HEASR, hydroethanolic extract of *A. Smeathmannii* (DC.) root, HEASR4, 250 mg/kg ofhydroethanolic extract of *A. Smeathmannii* (DC.) root, HEASR5, 500 mg/kg ofhydroethanolic extract of *A. Smeathmannii* (DC.) root, HEASR6, 1000 mg/kg ofhydroethanolic extract of *A. Smeathmannii* (DC.) root, ROS, reactive oxygen species, *Acridocarpus smeathmannii* (DC.) root, Subacute toxicity, Subchronic toxicity, Wistar rats, Safety study, Toxicology

## Abstract

Recent adverse herb reactions have stimulated interest documenting the safety profile of medicinal agents. Thus, subacute and subchronic oral toxicity of the hydroethanolic extract of *Acridocarpus smeathmannii* root (HEASR) in Wistar rats was investigated. In the 28 and 90-day subacute and subchronic toxicity tests, sixty-four rats (n = male: female = 1:1 = 32) were divided into four of eight/group and ninety-six (n = male: female = 1:1 = 48) into twelve/group respectively. Distilled water (10 mL/kg) or HEASR4, HEASR5 and HEASR6 (250, 500 and 1000 mg/kg/day) respectively were administered via oral gavage. Animals were killed humanely 24 h after the last administration. Using standard methods, acute oral toxicity dose of HEAR (2000 mg/kg) was non-lethal in rodents. Subacute administration of HEASR6 increased total bilirubin (p < 0.05) in female rats. HEASR moderately altered both haematological and biochemical indices in rats. HEASR6 administration reduced ovary weight in both studies while follicle stimulating hormone level in male was reduced at all doses used. HEASR modulated lipid peroxidation, sperm quality and elevated cyclooxygenase-2 levels in rats. Histology revealed gastritis and congestions in vital organs. The low-observed adverse effect level for HEASR was below 250 mg/kg for both sexes. Overall, HEASR demonstrated inherent toxicity evidenced by our current findings. The exaggeration of its folklore medicine applications calls for cautions.

## Graphical abstract

**Figure:**
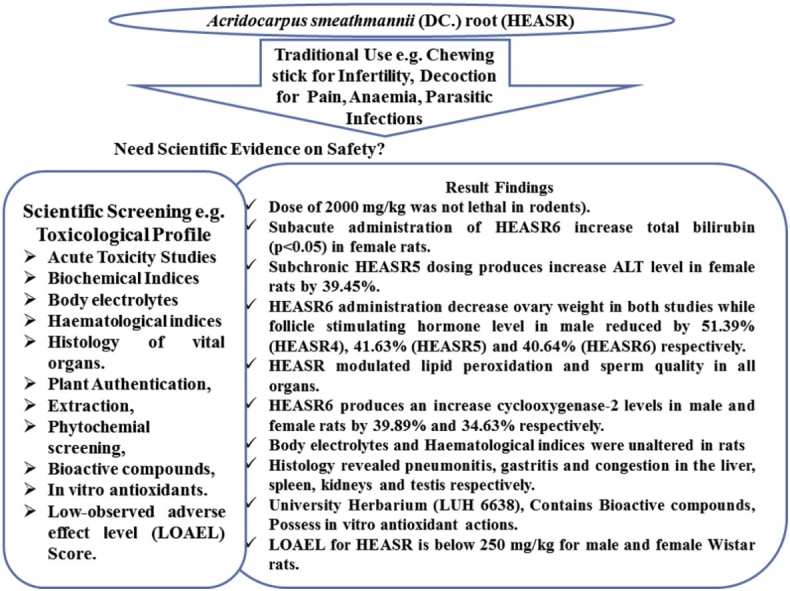

## 1 Introduction

Medicinal plants or products form an important part of our everyday life. The use of plants or their constituents for foods and medicines is as old as man. Reports have shown that over 70% of Africans or Asians depend on natural product medicines [1]. This is because they can easily be obtained, prepared and often attract a low cost. Several goals for using plants as sources of therapeutic agents have been identified and updated [2]. These include isolation of bioactive compounds, structural elucidation of lead compounds for development into drug molecules that would serve as pharmacologic tools and or whole plant or part of it as a herbal remedy [3,4]. Plants synthesize a variety of metabolites that form complex compounds that may be benefitial or harmful to mankind. Most of the developed nations exert certain levels of regulations and have developed reliable strategies for the monitoring of safety and standardization of these products while providing quality assurance for any of such natural substance [5,6]. However, many traditional and complementary medicine practitioners often refute the WHO certification scheme to regulate the quality of medicinal products [1]. This explains why there exist divergent opinions on the various applications of medicinal herbs [2]. Also, this constitutes a setback against the scientific justification of folklore medicines applications [7,8]. In order to ensure safety, the scientific community has birthed three notions. Firstly, there must be a study to show safety profiles of any compound/product that is claimed to be beneficial to a living organism. The second is to assess the chemical constituents of the traditional medicinal agent. And lastly is to set the guidelines to investigate the proposed folklore application which is a step towards drug development and discovery. Thus, every medicinal plant or product is being sought for, regarding the verification for public acceptance and consequently the necessity of toxicological reports [9].

One of the most difficult adverse events recently documented stem from intoxications associated with the use of herbal medicines and this has elicited concerns [10,5,11]. On the other hand, an assessment using appropriate tools for ascertaining herbal toxicity in a number of cases has failed to show any causal effects or has indicated only weak causal relationship [12,13]. More so, misuse of herbal concoctions, herb-drug interactions and poor pharmacovigilance surveys for medicinal products continues to be a challenge despite the availability of causality assessment tests already recruited to most developing countries [11,12]. It has also been reported that adverse herb reactions often overlap due to inherent toxic effects of herbal medicine and toxicities induced by handlings or during preparations [9,12].

Various government agencies have continued to provide information on herbs including use patterns, toxicity information, clinical trial data, and review of reported side effects from herbal medicine use. Studies have linked several effects of medicinal products to an antioxidant system that help quench free radicals of different forms which are constantly generated for the specific metabolic requirement in the body. Reports from animal studies in respect to economic importance, toxicological effects and herb-drug interactions for commonly used herbal medicines such as ginkgos, aloe vera, ginsengs, milk thistle and turmerics amongst others have been documented [14,15]. However, despite the efforts to improve drug discovery and development, only few medicinal plants have been explored and screened for toxicological actions.

The aforementioned facts necessitate the need to assess the toxicological profile of *Acridocarpus smeathmannii* (DC.) Guill. & Perr. (Malpighiaceae), a well-known tropical African plant routinely used alone or together with other herbs to prepare concoctions [16] for the management of different ailments including infertility, anaemia, pain and some cutaneous as well as subcutaneous parasitic infections [[17], [18], [19]]. We recently reported the aphrodisiac potentials and reproductive functions [20]. We also showed that *A. smeathmannii* is most abundant in bioactive compounds, including octadecanoic acid ethyl ester, docosenoic acid, amongst others. Currently, study on safety profile of the plant extract is lacking. Therefore in the present study, we evaluated the subacute and subchronic toxicological effects of *A. smeathmannii* in Wistar rats of both sexes.

## 2 Materials and method

### 2.1 Chemicals

All chemicals and reagents used were of analytical grade.

### 2.2 Preparation of plant extract

Fresh *A. smeathmannii* roots were purchased from Akinmorin Sabo, old Oyo State, Nigeria in June 2016. Authentication was done at the University of Lagos Herbarium (Reference no: LUH 6638). The extract was prepared and reconstituted as described elsewhere [20].

### 2.3 Experimental animals

Male and female Wistar albino rats were obtained from a commercial private colony in Badagry, Lagos State, Nigeria and housed at ambient temperature (22 ± 3 °C) and humidity with a 12-hour light-dark schedule within the Laboratory Animal Centre of the College of Medicine, University of Lagos, Nigeria. Rats were fed with rat pelleted diet (Grower Mash, Oyo State, Nigeria). Water was made available *ad* libitum. The College of Medicine, University of Lagos Health Research and Ethics Committee approved the experimental protocols (CMUL/HREC/09/18/424). The study conforms with the Kilkenny et al. [21] suggestions for reporting animal research and the U.S National Institutes of Health (NIH Publication No. 85-23, revised 1996) standards for studies involving experimental animals.

### 2.4 Acute oral toxicity test

HEASR of 200 and 2 ×, 4 ×, 8 × and 10 × 200 mg/kg were administered to mice via the oral gavage. Water was supplied *ad* libitum and distilled water was given to the control. Mice were observed for behavioral changes post-treatment. Behavioural modifications and death were scored immediately after treatment and hourly. In addition, acute oral toxicity study using limit dose test of Up and Down Procedure (2002) was conducted per OECD/OCDE Test Guidelines on Acute Oral Toxicity under a computer-guided Statistical Programme- AOT425statPgm, No 420 (2002) in rats, at a limit dose of 2000 mg/kg and 4000 mg/kg body weight per oral route and default of Sigma at 0.5. Two groups of five rats per group of male young adult Wistar rats were systemically selected out of a population of 20 Wistar rats (8–12 weeks old) by systematic randomization techniques. The population sample was selected such that the weight differences do not exceed ±10% of the mean initial weight of the sample population. The rats were fasted overnight prior to dosing on each occasion. A rat was picked at a time, weighed and dosed with equivalent 2000 mg/kg body weight of HEASR. After the extract administration, each rat was observed for the first 5 min after oral administration for signs of possible regurgitation and then kept in a cage for observation. Each rat was watched for every 15 min in the first 2 h after dosing, then every 30 min for the successive 6 h and then daily for the successive 38 h for the short-term outcome and the remaining 14 days for the long-term possible lethal outcome. All animals were monitored as aforementioned with individual records being maintained for each rat.

### 2.5 Experimental design and treatment

In subacute toxicity testing, sixty-four (68) (n = male: female = 1:1 = 32) Wistar rats were divided into four groups of eight animals/group while another ninety-six (96) (n = male: female = 1:1 = 48) were divided into twelve animals/group for subchronic study. Group 1 (control) received distilled water (10 mL/kg) while groups 2, 3 ad 4 were administered 250 mg/kg, 500 mg/kg and 1000 mg/kg of HEASR respectively.

### 2.6 Hematological assessments

Hematological assessments were done using a fully automated haematology analyzer (Pentra-XL 80, Horiba ABX, USA).

### 2.7 Analysis of sperm characteristics and morphology

The testes were carefully removed from each rat and analysed at room temperature using one epididymis of each rat by incising through the caudal epididymis to liberate its spermatozoa into the saline solution. The process of sperm characterizations followed the methods of Kale and Awodele [9].

### 2.8 Reduced Glutathione determination and lipid peroxidation assay

Reduced glutathione (GSH) and lipid peroxidation levels were estimated following the methods of Beutler et al. [22] and Varshney and Kale [23] respectively.

### 2.9 Biochemical assays

The liver and renal biomarker enzymes, proteins, and lipid profiles were assessed using commercial kits obtained from Randox Laboratories Ltd. (Crumlin, UK) and following procedures described by the manufacturer.

### 2.10 Serum electrolytes

A flame photometer (Sherwood, Model 410) was used to analyse some vital serum electrolytes including sodium, potassium, chloride ion and total calcium ions respectively.

### 2.11 Necropsy

The liver, kidney, testis, epididymis, brain, prostate, lung, spleen, pancreas, stomach, heart, and ovaries was carefully removed, weighed (in grams per kilogram body weight) and fixed in 10% formol saline, dehydrated in graded alcohol and embedded in paraffin. Other processes of sectioning, mounting and counter-staining with hematoxylin and eosin (H&E) histopathologic examinations were performed. Both serum and homogenized organs were used for biochemical analysis.

### 2.12 Cyclooxygenase-2 activity ELISA assay

An enzyme-linked immunosorbent assay kit (ELISA) was used to evaluate cyclooxygenase-2 (COX-2) activity in serum. Triplicate samples were tested twice per plate (intra assay: CV < 8% and inter-assay: CV < 10%) and expressed as Units/l [24]. Briefly, the optical density of each well was determined according to the manufacturer’s instructions.

### 2.13 Statistics

Differences between groups were determined by one-way analysis of variance (ANOVA) using Statistical Package for Social Sciences (SPSS, version 20.0) software for windows and Post hoc test for intergroup using the least significant difference, followed by Dunnett’s test. Significance was considered at p < 0.05. All results were expressed as the mean ± standard error of the mean.

## 3 Results

### 3.1 Acute oral toxicity test

There was no mortality observed during 24 h post-treatment at 2000 mg/kg, although, behavioral as well as morphological changes were marked in mice that received 4000 mg/kg HEASR and above orally. The animals showed mild to severe hyperactivity, scratching of the lower jaw, assisted rearing and weakness in at least 5 animals in the first 2 h. However, the effects diminished completely by day 14 post-administration.

### 3.2 Subacute and subchronic toxicity results

#### 3.2.1 Liver function test

**Fig. 1:**
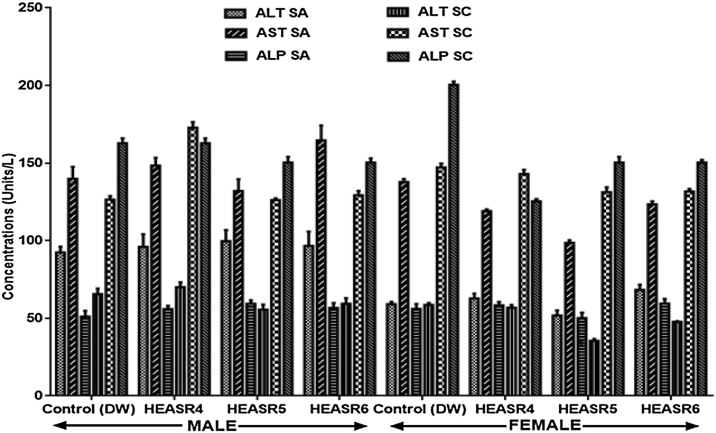
Effect of HEASR on liver function in enzymes in normal male Wistar rats. SA: Subacute, SC: Subchronic, AST: Aspartate aminotransferase, ALT: Alanine aminotransferase, ALP: Alkaline Phosphatase. Results are expressed as mean ± S.E.M. n: total number per group. (SA) = 8, n (SC) = 12. ^*^p < 0.05 or ^**^p < 0.01 when compared with control distilled water (DW, 10 mL/kg) group. HEASR4: 250 mg/kg, HEASR5: 500 mg/kg, HEASR6: 1000 mg/kg, HEASR: hydroethanolic extract of *Acridocarpus smeathmannii* root.

In the subacute and subchronic administrations, in male Wistar rats (Fig. 1), hepatic function enzymes were unaltered (p > 0.05) by HEASR (250, 500 and 1000 mg/kg) doses. Similar results were obtained for the female rats except for the ALT level that was slightly elevated (p > 0.05) in rats administered 500 mg/kg HEASR by 39.45% when compared with control.

#### 3.2.2 Renal biomarkers

**Fig. 2:**
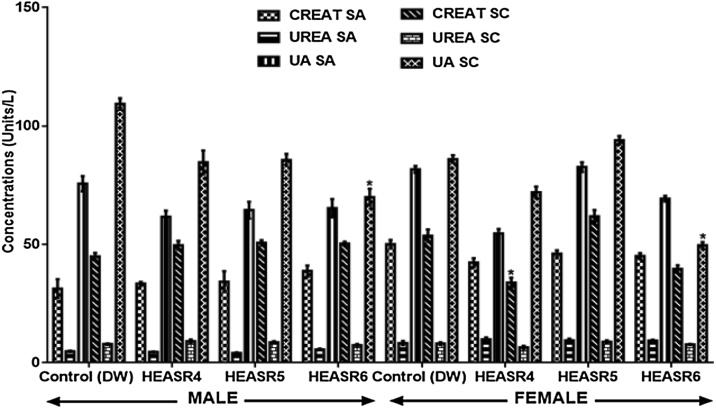
Effect of HEASR on creatinine, urea and uric acid levels in normal male Wistar rats. SA: Subacute, SC: Subchronic, CREAT: Creatinine, UREA: Blood Urea Nitrogen, UA: Uric Acid. Results are expressed as mean ± S.E.M. n: total number per group. n (SA) = 8, n (SC) = 12. Mortality: HEASR5 (8.3%, male), HEASR6 (16.67%, male), HEASR4 SC (8.3%, female), HEASR5 SC (16.7%, female) and HEASR6 SC (25%, female). ^*^p < 0.05 or ^**^p < 0.01 when compared with control (distilled water: DW, 10 mL/kg) group. Mortality: HEASR6 SA = 2. HEASR4: 250 mg/kg, HEASR5: 500 mg/kg, HEASR6: 1000 mg/kg, HEASR: hydroethanolic extract of *A. smeathmannii* root.

The creatinine, urea and uric acid levels did not change in male and female rats administered 250, 500 and 1000 mg/kg of HEASR respectively (Fig. 2). Also, in the subchronic administrations, creatinine and urea in male and female treated rats were not different from those of controls. However, the highest dose used in this study, 1000 mg/kg HEASR, lowered uric acid level in male rats by 35.97% and in the female by 42.24% (p < 0.05) respectively.

#### 3.2.3 Protein assays

**Fig. 3:**
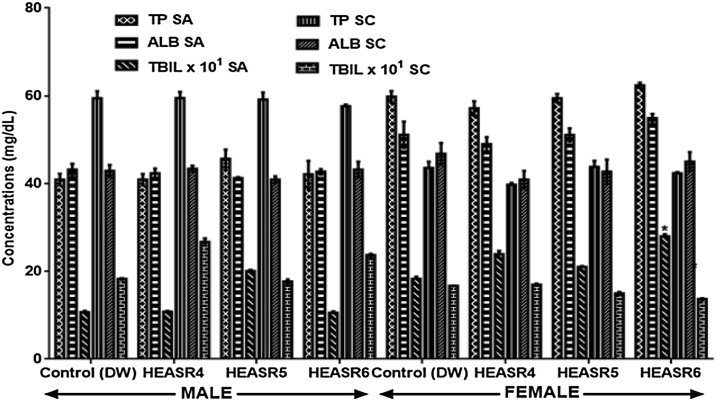
Effect of HEASR on serum total protein, albumin and total bilirubin in normal female Wistar rats. SA: Subacute, SC: Subchronic, TP: Total protein, ALB: Albumin, TBIL: total bilirubin. Results are expressed as mean ± S.E.M. n: total number per group. n (SA) = 8, n (SC) = 12. Mortality: HEASR5 (8.3%, male), HEASR6 (16.67%, male), HEASR4 SC (8.3%, female), HEASR5 SC (16.7%, female) and HEASR6 SC (25%, female). ^*^p < 0.05 or ^**^p < 0.01 when compared with control (distilled water: DW, 10 mL/kg) group. HEASR4: 250 mg/kg, HEASR5: 500 mg/kg, HEASR6: 1000 mg/kg, HEASR: hydroethanolic extract of *A. smeathmannii* root.

In male rats, 250 mg/kg, 500 mg/kg, and 1000 mg/kg did not alter serum total protein (TP), albumin (ALB) and total bilirubin (TBIL) levels as shown in Fig. 3. Similarly, in the subacute treated female rats, TP and ALB levels were unaltered at the low, medium and highest doses used in this study. But, TBIL increased by 53.01% (p < 0.05) in female rats that received HEASR6 when compared with control, whereas subchronic dosing for 90 days did not alter TP. ALB and TBIL levels in rats.

#### 3.2.4 Lipid profiles

**Fig. 4:**
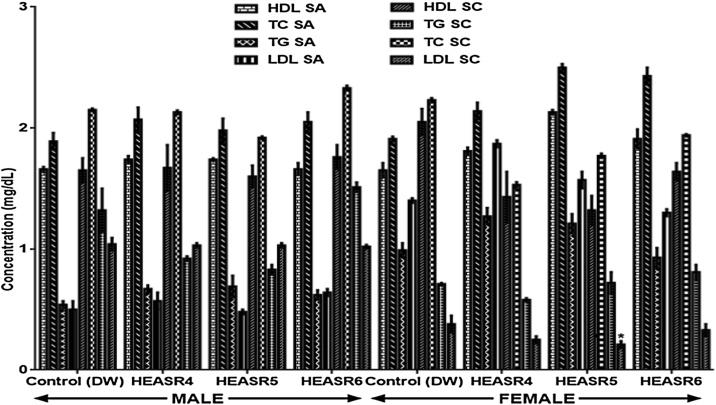
Effect of HEASR on lipid profiles levels in normal male Wistar rats. SA: Subacute, SC: Subchronic, HDL: High density lipoprotein, LDL: Low density lipoprotein, TC: Total cholesterol, TG: Triglyceride. Results are expressed as mean ± S.E.M. n: total number per group. n (SA) = 8, n (SC) = 12. Mortality: HEASR5 (8.3%, male), HEASR6 (16.67%, male), HEASR4 SC (8.3%, female), HEASR5 SC (16.7%, female) and HEASR6 SC (25%, female). ^*^p < 0.05 or ^**^p < 0.01 when compared with control (distilled water: DW, 10 mL/kg) group. HEASR4: 250 mg/kg, HEASR5: 500 mg/kg, HEASR6: 1000 mg/kg, HEASR: hydroethanolic extract of *A. smeathmannii* root.

Following a subacute treatment in normal male rats (Fig. 4), the serum HDL, TC, TG and LDL levels were unaltered in the treated rats. However, in the subacute female group, HEASR5 produce an elevated (p < 0.05) HDL level in rats by 40%. There was no change observed in TC and TG levels of female animals administered HEASR4, HEASR5, and HEASR6 respectively. More so, in subchronic treatment, both male and female rats that received HEASR4, HEASR5 and HEASR6 respectively showed no alteration in TC, TG and HDL levels respectively. Additionally, HEASR5 lowered LDL level by 44.74% when compared with control.

#### 3.2.5 Serum electrolytes

**Fig. 5:**
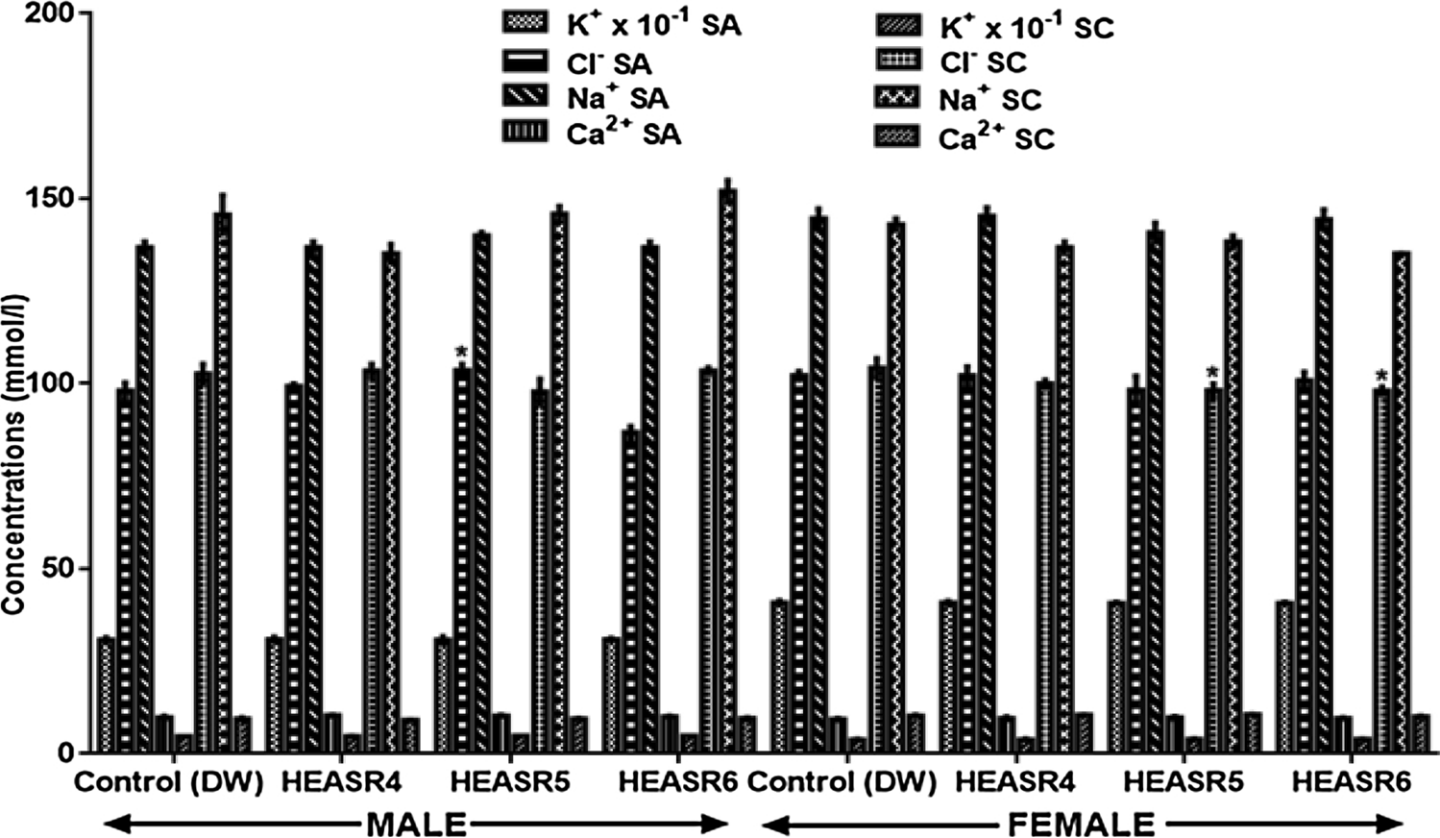
Effect of HEASR on body electrolytes in normal male Wistar rats. SA: Sub-acute, SC: Subchronic. K^+^: Potassium ion, Cl^−^: Chlorine ion, Na^+^: Sodium ion, Ca^2+^: Calcium ion. Results are expressed as mean ± S.E.M. n: total number per group. n (SA) = 8, n (SC or CH) = 12. Mortality: HEASR5 (8.3%, male), HEASR6 (16.67%, male), HEASR4 SC (8.3%, female), HEASR5 SC (16.7%, female) and HEASR6 SC (25%, female). ^*^p < 0.05 or ^**^p < 0.01 when compared with control (distilled water: DW, 10 mL/kg) group. HEASR4: 250 mg/kg, HEASR5: 500 mg/kg, HEASR6: 1000 mg/kg, HEASR: hydroethanolic extract of *Acridocarpus smeathmannii* root.

In both subacute and subchronic HEASR treatments of 250, 500 and 1000 mg/kg respectively, no change (p > 0.05) in electrolyte levels were observed in all the treated rats when compared with normal distilled water control group (Fig. 5).

#### 3.2.6 Haematological parameters

In subacute haematological assessment in male Wistar rats, HEASR6 elevated (p < 0.05) WBC and GRAN levels by 26.73% and 71.5% respectively when compared with control (Table 1). Similarly, in the subacute treated female rats, HEASR5 and HEASR6 reduced WBC and GRAN by 20.21%, 50.09% and 41.01%, 72.62% respectively. Also, LYMP and MID% levels were increased (p < 0.05) following an administration of HEASR4 (141.43%, 65.24%), HEASR5 (101.43% and 93.60%), and HEASR6 (72.27%, 99.11%) respectively. Similarly, MID increased by 84.21% in the HEASR5 treated rats. Given subchronic treatment, in male rats, an increase (p < 0.05) in LYMP level was obtained in HEASR4 (157.90%) and HEASR6 (170.65%) respectively. Also, GRAN% was reduced in HEARS4 and HEASR6 treated rats by 125.30% and 48.80% respectively. However, in the female rats, subchronic treatment with HEASR4, HEASR5 and HEASR6 reduced WBC and LYMP by 41.75%, 26.33%; 42.29%, 48.78%; and 50%, 53.66% respectively (Table 2).

**Table 1:** Subacute and Subchronic Effect of HEASR on haematological indices in normal male rats.

	Control SA	HEASR4 SA	HEASR5 SA	HEASR6 SA	Control SC	HEASR4 SC	HEASR5 SC	HEASR6 SC
WBC	5.50 ± 0.40	4.50 ± 1.20	4.87 ± 1.22	6.97 ± 2.28	5.60 ± 0.26	5.17 ± 0.22	5.97 ± 0.52	6.77 ± 0.76
LYMPH	2.75 ± 0.03	2.03 ± 0.67	1.43 ± 0.09^*^	2.87 ± 0.78	0.57 ± 0.27	1.47 ± 0.62^*^	0.77 ± 0.58	1.20 ± 0.81^*^
MID	0.75 ± 0.26	0.63 ± 0.17	0.63 ± 0.87	0.67 ± 0.24	2.50 ± 0.21	2.50 ± 0.25	2.33 ± 0.35	3.13 ± 0.09
GRAN	2.00 ± 0.12	1.83 ± 0.67	1.80 ± 0.50	3.43 ± 1.36^*^	2.53 ± 0.60	5.70 ± 4.68^*^	2.87 ± 0.52	2.43 ± 0.32
LYMPH%	50.10 ± 2.94	44.80 ± 7.72	35.53 ± 3.36^*^	43.50 ± 4.56	10.63 ± 1.67	28.77 ± 1.02^*^	11.80 ± 1.07	16.27 ± 1.98^*^
MID%	13.65 ± 3.72	14.87 ± 0.41	28.50 ± 1.29	19.50 ± 0.61	44.90 ± 4.73	48.47 ± 3.99	38.50 ± 4.56	47.57 ± 5.60
GRAN%	36.25 ± 0.78	40.33 ± 7.92	35.97 ± 4.69	47.00 ± 4.76	44.47 ± 2.70	22.77 ± 3.21^*^	49.70 ± 1.98	36.17 ± 4.73
HGB	13.50 ± 0.23	13.10 ± 0.75	12.50 ± 0.45	13.20 ± 0.29	13.80 ± 0.31	13.80 ± 0.15	14.53 ± 0.62	14.10 ± 0.06
RBC	6.88 ± 0.20	6.41 ± 0.50	6.70 ± 0.30	6.65 ± 0.26	6.63 ± 0.35	6.15 ± 0.58	7.08 ± 0.71	6.85 ± 0.39
HCT	42.30 ± 0.17	40.40 ± 2.89	39.10 ± 2.22	40.87 ± 1.25	48.33 ± 2.09	44.73 ± 1.89	48.93 ± 4.14	47.67 ± 1.07
MCV	61.80 ± 2.08	63.47 ± 4.22	58.37 ± 0.81	61.60 ± 0.50	73.20 ± 3.03	73.67 ± 4.62	69.33 ± 1.45	75.90 ± 6.45
MCH	19.60 ± 0.23	20.50 ± 0.82	18.60 ± 0.21	19.80 ± 0.42	20.83 ± 0.78	22.77 ± 2.10	20.73 ± 1.36	22.33 ± 1.51
MCHC	31.85 ± 0.66	32.50 ± 0.93	32.00 ± 0.83	32.27 ± 0.41	28.57 ± 0.64	30.90 ± 0.98	29.87 ± 1.39	29.53 ± 0.54
RDWCV	16.25 ± 0.55	17.33 ± 0.49	15.87 ± 0.85	15.50 ± 1.40	17.27 ± 0.66	17.77 ± 0.33	17.13 ± 0.72	16.40 ± 0.46
RDWSD	34.20 ± 2.14	34.90 ± 1.55	31.90 ± 2.29	33.43 ± 1.89	39.33 ± 1.10	39.57 ± 0.27	38.33 ± 1.77	38.13 ± 0.23
PLT	742.50 ± 2.59	678.33 ± 5.90	568.00 ± 3.70^*^	750.00 ± 3.87	831.33 ± 2.67	786.67 ± 6.64	889.33 ± 2.54	849.67 ± 4.34
MPV	8.00 ± 0.40	8.07 ± 0.17	7.40 ± 0.12	7.40 ± 0.15	9.30 ± 0.23	9.97 ± 0.58	9.33 ± 0.20	9.27 ± 0.27
PDW	15.20 ± 0.12	15.43 ± 0.12	15.70 ± 0.17	15.23 ± 0.15	15.80 ± 0.06	15.70 ± 0.10	15.67 ± 0.15	15.67 ± 0.03
PCT	0.59 ± 0.03	0.55 ± 0.04	0.42 ± 0.02^*^	0.56 ± 0.04	0.55 ± 0.01	0.62 ± 0.04	0.48 ± 0.02	0.67 ± 0.01^*^

SA: Subacute, SC: Subchronic. HEASR: Hydroethanolic extract of *Acridocarpus smeathmannii* root. HEASR4: 250 mg/kg; HEASR5: 500 mg/kg. HEASR6: 1000 mg/kg. Data are expressed as mean ± SEM. n (SA) = 8, n (SC) = 12. Mortality: HEASR5 (8.3%, male), HEASR6 (16.67%, male), HEASR4 SC (8.3%, female), HEASR5 SC (16.7%, female) and HEASR6 SC (25%, female). ^**^p < 0.05 or ^**^p < 0.001 when compared with control distilled water (10 mL/kg) group. WBC x 10^3^ (/mL) White Blood cell, LYMPH x 10^3^ (/mL) Lymphocyte, MID (%): Minimum Inhibitory Dilution, GRAN (%)`: Granulocytes, HGB (g/dL): Hemoglobin, RBC x 10^6^ /mL: Red Blood Cell, HCT (%): Hematocrit, MCV (fL): Mean Corpuscular Volume, MCH (pg/dL): Mean Corpuscular Hemoglobin, MCHC (g/dL): Mean Corpuscular Hemoglobin Concentration, RDWCV (%): Red Blood Cell Volume Distribution Width-CV, RDWSD (%): Red Blood Cell Volume Distribution Width-SD, PLT: Platelet, MPV (fL): Mean Platelet Volume, PDW: Plate Volume Distribution Width, PCT (%): Plateletcrit.

**Table 2:** Subacute and Subchronic Effect of HEASR on haematological indices in normal female rats.

	Control SA	HEASR4 SA	HEASR5 SA	HEASR6 SA	Control SC	HEASR4 SC	HEASR5 SC	HEASR6 SC
WBC	12.417 ± 0.15	12.98 ± 0.17	9.9.1 ± 0.21^*^	6.19 ± 0.32^*^	7.33 ± 1.74	4.27 ± 0.57^*^	5.40 ± 0.10^*^	4.23 ± 0.13^*^
LYMPH	2.10 ± 0.40	5.07 ± 0.17^*^	3.47 ± 0.69^*^	4.23 ± 0.93^*^	4.10 ± 0.06	2.10 ± 0.40^*^	2.05 ± 0.22^*^	1.90 ± 0.06^*^
MID	3.21 ± 0.370	5.91 ± 0.17^*^	4.25 ± 0.26	3.3 ± 0.29	1.30 ± 0.05	0.73 ± 0.09	0.90 ± 0.10	0.70 ± 0.06^*^
GRAN	89.97 ± 9.72	61.60 ± 3.87	53.07 ± 3.14^*^	24.63 ± 2.29^*^	1.93 ± 0.34	1.43 ± 0.09	1.93 ± 0.20	1.63 ± 0.09
LYMPH%	14.71 ± 0.87	17.80 ± 3.58	13.67 ± 2.14	18.97 ± 1.49^*^	55.40 ± 1.04	48.30 ± 0.61	47.60 ± 2.75	44.67 ± 1.52
MID%	25.93 ± 1.04	50.20 ± 2.75^*^	44.67 ± 2.12^*^	51.63 ± 2.99^*^	17.33 ± 0.91	17.70 ± 0.36	16.07 ± 2.05	15.97 ± 0.43
GRAN%	72.60 ± 1.42	42.00 ± 2.20^*^	51.67 ± 3.25	29.40 ± 2.88^**^	26.60 ± 1.50	34.00 ± 0.61	36.33 ± 4.56^*^	39.37 ± 1.09^*^
HGB	9.20 ± 1.14	8.87 ± 0.99	7.17 ± 2.63	7.97 ± 1.43	13.83 ± 0.84	13.53 ± 0.12	13.67 ± 0.46	13.13 ± 0.28
RBC	1.54 ± 0.11	1.83 ± 0.20	1.76 ± 0.24	1.68 ± 0.65	6.96 ± 0.33	6.74 ± 0.11	7.02 ± 0.21	6.18 ± 0.13
HCT	22.73 ± 2.51	23.60 ± 2.80	19.13 ± 3.94^*^	21.63 ± 4.84	44.30 ± 1.01	40.93 ± 0.27^*^	43.10 ± 0.60	37.03 ± 0.84
MCV	157.90 ± 4.10	167.07 ± 4.08	165.50 ± 8.57	144.03 ± 2.04	63.80 ± 0.40	60.77 ± 0.52^*^	61.57 ± 1.03	60.03 ± 0.39
MCH	63.57 ± 0.96	62.70 ± 2.19	62.50 ± 4.45	55.30 ± 1.29	19.80 ± 0.29	19.27 ± 0.42	19.40 ± 0.38	19.57 ± 0.19
MCHC	40.30 ± 0.80	37.60 ± 0.47	37.80 ± 1.17	37.53 ± 2.27	31.13 ± 0.43	31.80 ± 0.46	31.63 ± 0.74	32.73 ± 0.52
RDWCV	12.57 ± 1.15	10.67 ± 1.28	12.57 ± 0.58	12.70 ± 3.11	17.20 ± 0.44	16.73 ± 0.33	15.80 ± 0.95	14.60 ± 0.32
RDWSD	75.87 ± 7.37	78.37 ± 1.11	84.57 ± 4.75	57.23 ± 4.12	36.83 ± 0.91	33.90 ± 0.87	34.20 ± 1.97	29.67 ± 0.87
PLT	78.00 ± 2.50	50.67 ± 1.29	58.00 ± 5.51	231.33 ± 1.84	793.67 ± 1.26	882.00 ± 1.75	774.67 ± 1.48	746.00 ± 1.67
MPV	7.27 ± 0.15	6.90 ± 0.80	7.77 ± 0.52	8.03 ± 1.09	8.23 ± 0.19	7.77 ± 0.17	7.53 ± 0.33	7.73 ± 0.17
PDW	16.80 ± 0.21	16.93 ± 0.23	16.97 ± 0.26	16.53 ± 0.55	15.37 ± 0.03	15.43 ± 0.03	15.20 ± 0.15	15.50 ± 0.21
PCT	0.06 ± 0.01	0.03 ± 0.01	0.05 ± 0.01	0.02 ± 0.02^**^	0.65 ± 0.01	0.48 ± 0.19	0.58 ± 0.05	0.57 ± 0.04

SA: Subacute, SC: Subchronic. HEASR: Hydroethanolic extract of *Acridocarpus smeathmannii* root. HEASR4: 250 mg/kg; HEASR5: 500 mg/kg. HEASR6: 1000 mg/kg. Data are expressed as mean ± SEM. n (SA) = 8, n (SC) = 12. Mortality: HEASR5 (8.3%, male), HEASR6 (16.67%, male), HEASR4 SC (8.3%, female), HEASR5 SC (16.7%, female) and HEASR6 SC (25%, female). ^**^p < 0.05 or ^**^p < 0.001 when compared with control distilled water (10 mL/kg) group. WBC x 10^3^ (/mL) White Blood cell, LYMPH x 10^3^ (/mL) Lymphocyte, MID (%): Minimum Inhibitory Dilution, GRAN (%)`: Granulocytes, HGB (g/dL): Hemoglobin, RBC x 10^6^ /mL: Red Blood Cell, HCT (%): Hematocrit, MCV (fL): Mean Corpuscular Volume, MCH (pg/dL): Mean Corpuscular Hemoglobin, MCHC (g/dL): Mean Corpuscular Hemoglobin Concentration, RDWCV (%): Red Blood Cell Volume Distribution Width-CV, RDWSD (%): Red Blood Cell Volume Distribution Width-SD, PLT: Platelet, MPV (fL): Mean Platelet Volume, PDW: Plate Volume Distribution Width, PCT (%): Plateletcrit.

#### 3.2.7 Sperm morphology

**Fig. 6:**
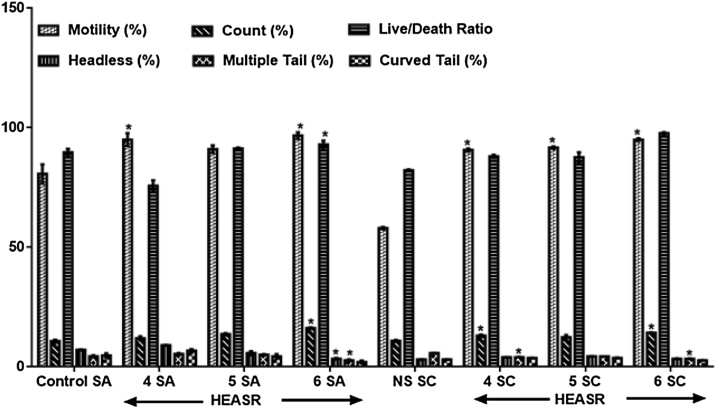
Effect of HEASR on sperm morphological characteristics in normal male Wistar rats. SA: Subacute, SC: Subchronic. Calcium ion. Results are expressed as mean ± S.E.M. n: total number per group. n (SA) = 8, n (SC) = 12. Mortality: HEASR5 (8.3%, male), HEASR6 (16.67%, male), HEASR4 SC (8.3%, female), HEASR5 SC (16.7%, female) and HEASR6 SC (25%, female). ^*^p < 0.05 or ^**^p < 0.01 when compared with control (distilled water: DW, 10 mL/kg) group. HEASR4: 250 mg/kg, HEASR5: 500 mg/kg, HEASR6: 1000 mg/kg, HEASR: hydroethanolic extract of *A. smeathmannii* root.

In the subacute treated group, HEASR4, HEASR5 and HEASR6 increased sperm motility by 12.81% (p > 0.05), 17.76% (p > 0.05) and 19.83% (p < 0.05) respectively (Fig. 6). The HEASR6 administered to rats increased significantly sperm counts by 50.75%. Also, there was increased headless sperm for low, medium and highest doses by 52.43%, 38.34%, and 57.13% respectively. In the subchronic administration, HEASR4, HEASR5, and HEASR6 show increase (p < 0.05) in sperm motility by 56.32%, 58.05%, and 63.79% respectively. And interestingly, multiple tails scoring in rats was lowered by 29.45% and 41.27% respectively in rats that received HEASR4 and HEASR6.

#### 3.2.8 Oxidative stress assessment

**Fig. 7:**
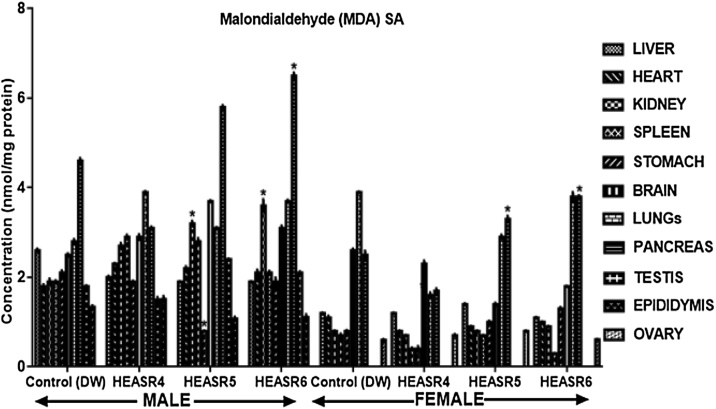
Effect of HEASR on lipid peroxidation in normal Wistar rats. SA: Subacute, MDA: Malondialdehyde. Results are expressed as mean ± S.E.M. n: total number per group. n (SA) = 8. ^*^p < 0.05 or ^**^p < 0.01 when compared with control (distilled water: DW, 10 mL/kg) group. HEASR4: 250 mg/kg, HEASR5: 500 mg/kg, HEASR6: 1000 mg/kg, HEASR: hydroethanolic extract of *Acridocarpus smeathmannii* root.

**Fig. 8:**
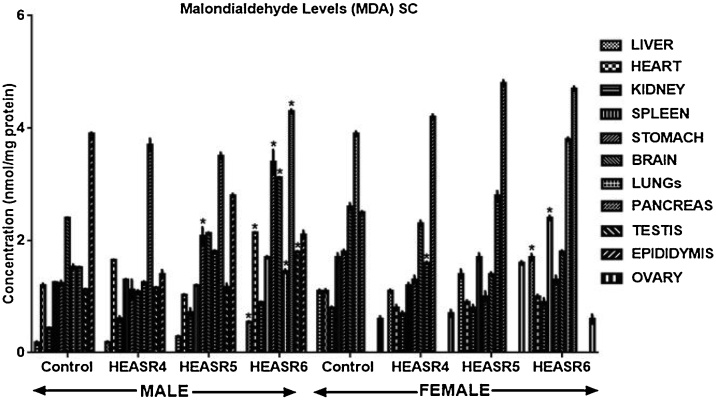
Effect of HEASR on lipid peroxidation in normal Wistar rats. SC: Subchronic, MDA: Malondialdehyde. Calcium ion. Results are expressed as mean ± S.E.M. n: total number per group. n (SC) = 12. Mortality: HEASR5 (8.3%, male), HEASR6 (16.67%, male), HEASR4 SC (8.3%, female), HEASR5 SC (16.7%, female) and HEASR6 SC (25%, female). ^*^p < 0.05 or ^**^p < 0.01 when compared with control (distilled water: DW, 10 mL/kg) group. HEASR4: 250 mg/kg, HEASR5: 500 mg/kg, HEASR6: 1000 mg/kg, HEASR: hydroethanolic extract of *Acridocarpus smeathmannii* root.

The effect of HEASR on lipid peroxidation (MDA) in normal male rats increased (p < 0.05) in the kidneys of HEASR5 and HEASR6 by 68.42% and 89.47% respectively following subacute administration (Fig. 7). In contrast, HEASR5 and HEASR6 decreased hepatic MDA levels by 26.92% each. Also, HEASR5 produced lowered (p < 0.05) MDA level in the stomach of the treated rats, whereas HEASR6 elevated pancreas MDA levels by 42.2%. In the subacute treated female rats, MDA level was unaltered in the spleen, brain, lungs; however, it increased in the pancreas by 32% (HEASR5) and 52% (HEASR6) respectively. In the subchronic administration, in male rats (Fig. 8), MDA increased (p < 0.05) in HEASR6 treated rats in the testis, liver, pancreas, and heart by 59.29%, 205%, 182.90%, and 78.33% respectively. More so, the MDA levels increased in the stomach of HEASR5 and HEASR6 treated rats by 67.74% and 174.19% respectively. But in the female rats, the subchronic effect of HEASR6 administration caused increased (p < 0.05) MDA levels in liver (54.54%) and spleen (41.18%). Further, HEASR5 decreased (p < 0.05) lungs MDA level by 58.97% when compared with control.

#### 3.2.9 Antioxidant assays

**Fig. 9:**
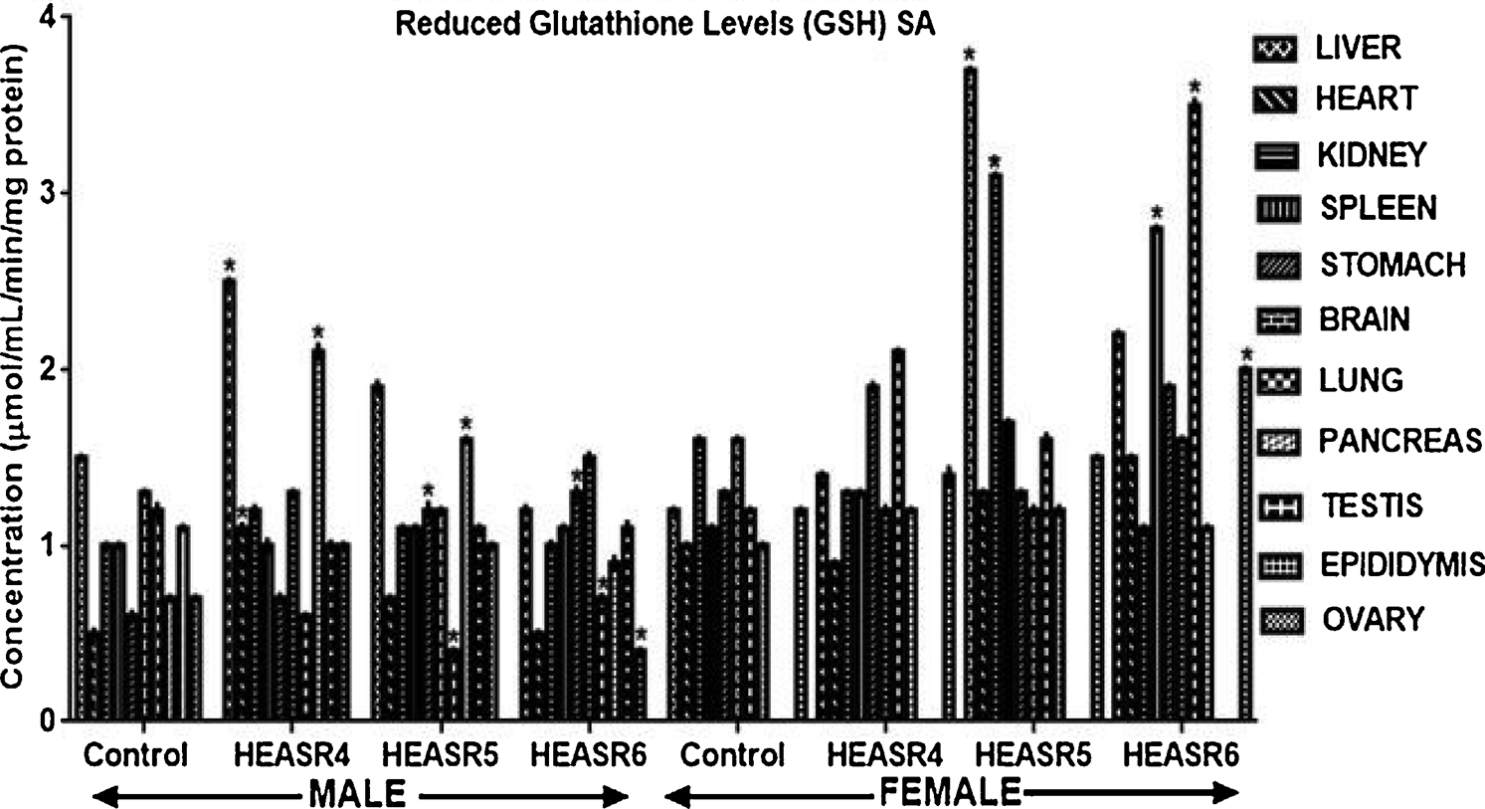
Effect of HEASR on reduced glutathione in normal Wistar rats. SA: Subacute. Results are expressed as mean ± S.E.M. n: total number per group. n (SA) = 8. ^*^p < 0.05 or ^**^p < 0.01 when compared with control (distilled water: DW, 10 mL/kg) group. HEASR4: 250 mg/kg, HEASR5: 500 mg/kg, HEASR6: 1000 mg/kg, HEASR: hydroethanolic extract of *Acridocarpus smeathmannii* root.

**Fig. 10:**
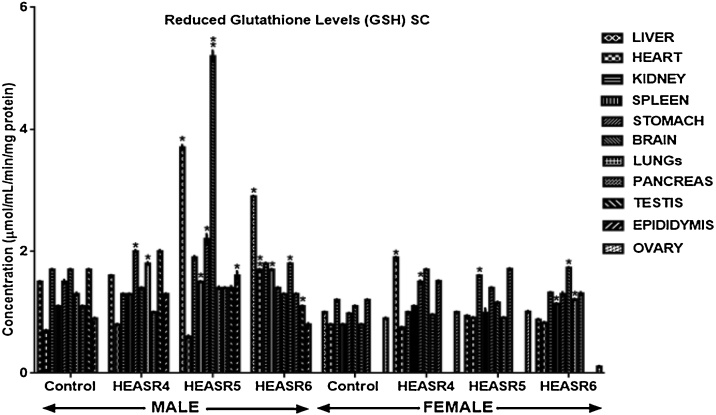
Effect of HEASR on reduced glutathione in normal Wistar rats. SC: Subacute. Results are expressed as mean ± S.E.M. n: total number per group. n (SC) = 12. Mortality: HEASR5 (8.3%, male), HEASR6 (16.67%, male), HEASR4 SC (8.3%, female), HEASR5 SC (16.7%, female) and HEASR6 SC (25%, female). ^*^p < 0.05 or ^**^p < 0.01 when compared with control (distilled water: DW, 10 mL/kg) group. HEASR4: 250 mg/kg, HEASR5: 500 mg/kg, HEASR6: 1000 mg/kg, HEASR: hydroethanolic extract of *Acridocarpus smeathmannii* root.

Results in Fig. 9 showed that administration of HEASR4 produced increase (p < 0.05) in GSH levels in the pancreas, liver, and heart by 200%, 66.67%, and 120% respectively in male rats. Also, elevated (p < 0.05) GSH levels were obtained in the pancreas and stomach of rats that received HEASR5 administration by 128.57% and 100% respectively, although, it decreased in the lungs by 66.67% when compared with control. In female subacute treated rats, hepatic and renal GSH levels were increased (p < 0.05) in rats administered HEASR5 by 208% and 93.75% respectively. Also, HEASR6 produced elevated GSH levels in the lungs, ovaries, and spleen by 191.67%, 66.67%, and 154.55% respectively. On subchronic treatment (Fig. 10), in male rats, HEASR4 administration increased (p < 0.05) GSH levels by 33.33% and 38.46% in the stomach and lungs respectively. Also, HEASR5 produced elevated (p < 0.05) GSH levels in the epididymis, spleen, stomach, and liver by 77.78%, 36.36%, 46.67%, and 146.67% respectively. Additionally, HEASR5 administration improved (p < 0.001) brain GSH level in rats by 205.88%. The highest dose used in this study, HEASR6 increased (p < 0.05) GSH level in the spleen. In the female rats, the administration of HEASR4 increased (p < 0.05) GSH levels in the liver (90%), brain (54.55%) and stomach (53.06%) respectively. Additionally, HEASR6 elevated GSH levels by 51.25%, 57.27 and 42.5% respectively in the lungs, brain, and spleen.

#### 3.2.10 Organ weight

**Fig. 11:**
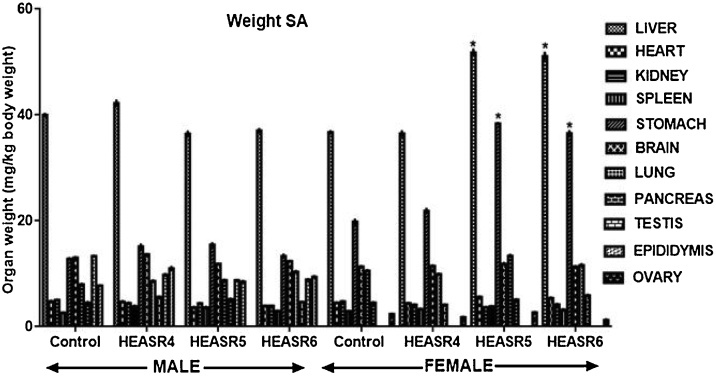
Effect of HEASR on organ weight relative to body weight of normal Wistar rats. SA: Subacute. Results are expressed as mean ± S.E.M. n: total number per group. n (SA) = 8. ^*^p < 0.05 when compared with control (distilled water: DW, 10 mL/kg) group. HEASR4: 250 mg/kg, HEASR5: 500 mg/kg, HEASR6: 1000 mg/kg, HEASR: hydroethanolic extract of *A. smeathmannii* root.

**Fig. 12:**
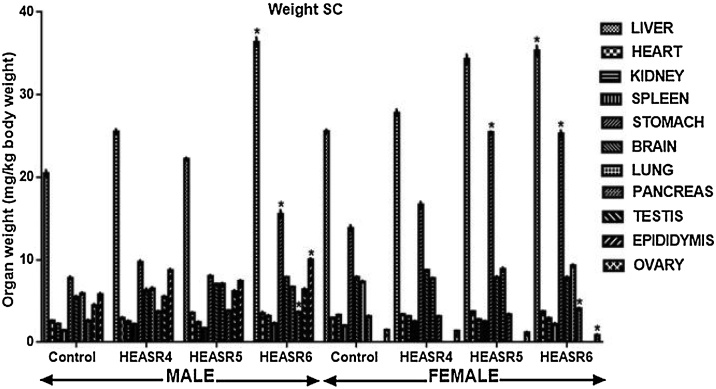
Effect of HEASR on organ weight relative to body weight of normal Wistar rats. SC: Subchronic. Results are expressed as mean ± S.E.M. n: total number per group. n (SC) = 12. Mortality: HEASR5 (8.3%, male), HEASR6 (16.67%, male), HEASR4 SC (8.3%, female), HEASR5 SC (16.7%, female) and HEASR6 SC (25%, female). ^*^p < 0.05 when compared with control distilled water (DW, 10 mL/kg) group. HEASR4: 250 mg/kg, HEASR5: 500 mg/kg, HEASR6: 1000 mg/kg, HEASR: hydroethanolic extract of *Acridocarpus smeathmannii* root.

The subacute effect of HEASR on organ weights relative to body weight in normal Wistar rats is presented in Fig. 11. The testis, epididymis, kidneys, spleen, pancreas, liver, heart, lungs and brain were unchanged in the after subacute administration in male rats. In the subacute treatment of female rats, however, HEASR6 reduced weight of ovaries by 47.72% when compared with control distilled water group. In contrast, HEASR5 and HEASR6 increased (p < 0.05) liver and stomach weights by 40.93%, 93.50% and 39.07%, 84.07% respectively. Spleen, kidneys, brain, and heart weight were not significantly altered in these rats. In the subchronic administration (Fig. 12), male Wistar rats that received HEASR6 showed an increase in epididymis, pancreas, stomach, and liver weights by 73.58%, 159.75%, 94.37%, and 77.77% respectively. Also, in subchronic treated female rats, HEASR5 increased stomach weight by 84.21% while HEASR6 also increased (p < 0.05) liver, stomach, and pancreas weights by 38.31%, 80.06%, and 30.16% respectively. In addition, HEASR6 reduced weight of the ovary weight by 41.61% in the treated rats.

#### 3.2.11 Hormonal Assessments

Subchronic effect of HEASR on reproductive hormone levels in normal male Wistar rats is presented in Table 3. There was a dose-dependent increase (p < 0.05) in testosterone levels in rats that received HEASR4, HEASR5 and HEASR6 by 84.75%, 184.75%, and 344.07% respectively when compared with control distilled water group. Also, HEASR6 elevated LH level by 39.68% in the treated rats. Contrastingly, FSH level was lowered in all the treated male groups by 51.39% (HEASR4), 41.63% (HEASR5) and 40.64% (HEASR6) respectively compared with control. Whereas in the female rats LH, FSH and oestrogen levels were increased in the rats that received HEASR4 by 182.04% (p < 0.001), 112.84% (p < 0.05) and 11.51% (p > 0.05) respectively. Further, HEASR6 elevated LH level by 198% (p < 0.001) while it decreased oestrogen level by 29.77%.

**Table 3:** Effect of hydroethanolic root of *Acridocarpus smeathmannii* extract on body hormones and COX-2 in serum of normal and treated rats using enzyme-linked immunosorbent assays.

	Control	HEASR4 (250 mg/kg)	HEASR5 (500 mg/kg)	HEASR6 (1000 mg/kg)
TST^x^	2.36 ± 0.07	4.36 ± 0.07^*^	6.72 ± 0.03^**^	10.48 ± 0.09^**^
LH^x^	25.53 ± 1.20	31.12 ± 1.10	34.62 ± 1.61	35.66 ± 1.11^*^
FSH^x^	351.05 ± 3.80	170.63 ± 3.21^*^	204.90 ± 2.40^*^	208.39 ± 3.50^*^
COX-2^x^	2.316 ± 0.12	2.280 ± 0.13	2.856 ± 0.12	3.240 ± 0.20
LH^y^	17.48 ± 0.20	49.30 ± 0.40^**^	20.63 ± 0.61	52.10 ± 0.20^**^
FSH^y^	230.42 ± 1.53	490.72 ± 1.50^*^	233.42 ± 1.24	237.42 ± 1.72
OEST^y^	79.25 ± 1.09	88.37 ± 0.54	80.50 ± 0.36	55.66 ± 0.18
COX-2^y^	3.216 ± 0.12	3.516 ± 0.12	4.14 ± 0.020	4.920 ± 0.16

HEASR: hydroethanolic extract of *Acridocarpus smeathmannii* root. Results are expressed as mean ± S.E.M. n = 6. Mortality: HEASR5 (8.3%, male), HEASR6 (16.67%, male), HEASR4 SC (8.3%, female), HEASR5 SC (16.7%, female) and HEASR6 SC (25%, female). ^*^p < 0.05 or ^**^p< 0.01 when compared with control distilled water group. “x” and “y” in superscripts represented “male” and “female” rats respectively. LH: Luteinizing hormone (mIU/L), FSH: Follicle stimulating hormone (ng/mL), ESTR: Estrogen (pg/mL), PROGES: Progesterone (ng/mL), COX−2: cyclooxygenase−2 (ng/mL).

#### 3.2.12 COX-2 assessment

The subchronic administration of HEASR6 produces elevated (p > 0.05) COX-2 levels in the treated male and female rats by 39.89% and 34.63% respectively (Table 3).

#### 3.2.13 Percentage mortality assessments

Animals were assessed for behavioral abnormalities and mortality during administration. There was no mortality during subacute dosing for both sexes. However, during subchronic treatment, mortality was recorded for rats that were administered HEASR5 (500 mg/kg) and HEASR6 (1000 mg/kg) both male and female as HEASR5 (8.3%, male), HEASR6 (16.67%, male), HEASR4 SC (8.3%, female), HEASR5 SC (16.7%, female) and HEASR6 SC (25%, female) respectively.

## 4 Discussion

Traditional and complementary medicine has been in use over many centuries. Also, its popularity and extensive use by a large number of people have challenged orthodox practices in several manners, although, traditional medicine still thrives to be officially to be recognized in few countries [19]. Lack of regulation is commonplace and has impacted negatively worldwide. This may be due to lack of scientific research data and adequate research methodology for evaluating medicinal products [10,19]. Thus, several herbs and their preparations have been labeled for toxicity and review worldwide [25,2,26]. Warnings have been issued by the Food and Drug Administration as regards the potential toxic effects of many commonly consumed medicinal plants and/or herbal preparations [25]. Toxicological risks of medicinal plants used in different parts of the world have been documented [27]. Safety testing is needed in order to popularize acceptance, standardize and/or regulate the market of herbal medicines currently being offered [9]. However, in recent times, scientists have inquire into scrutinizing the quantity and quality of the safety and efficacy potential acclaimed in folklore medicine in order to provide data to meet the criteria needed to support its use worldwide [[28], [29], [30], [31]].

*A. smeathmannii* is being used by a large population and is also present in some locally consumed polyherbal [17,19]. *A. smeathmannii* is available as a natural medicinal agent, but there is currently no dose regulation. However, safe dose for human consumption may be extrapolated from this current findings. Toxicity testing can be used to obtain information on the biologic activity of a chemical substance and gain insight into its mechanisms of action [30,32,31]. More so, toxicological evaluation studies are important aspects of drug development and for the extension of their therapeutic potentials [[8], [9], [10], [11], [12], [13], [14], [15], [16], [17], [18], [19], [20], [21], [22], [23], [24], [25], [26], [27], [28], [29], [30], [31]]. Thus, it helps pinpoint information on the adverse reactions which are potentially relevant to the substance being evaluated [33]. These potential effects may include behavioural, psychological, biochemical, morphological, neurological, metabolic, teratological, mutagenic and histological alterations [32]. In practice, the substance is administered orally or in some cases via parenteral route and then observed for conditions aforementioned. Both dose and duration approaches have helped in understanding toxicity evaluation as well as exposure risks [32,34,36,7]. While we reported some important bioactive compounds present in HEASR [20], neither HEASR nor any of its components have been evaluated for toxicity. Therefore, in the present study, we assessed the dose and time-dependent subacute and subchronic toxicological effects of HEASR in Wistar rats of both sexes.

We showed that there was no mortality in rats that received 2000 mg/kg HEASR orally indicating that it may be relatively safe for consumption upon acute administration at this dose. Also, since HEASR is usually administered via the oral route, this enabled a dose selection of 250 (1/8th), 500 (1/4th) and 1000 mg/kg (1/2th) (LD50 oral gavage > 2000 mg/kg). During subacute and subchronic administrations, hepatic function enzymes were unaltered in male rats that received HEASR (250, 500 and 1000 mg/kg) at all doses tested. Similar results ensued in the female rats. Assessment of hepatic biomarker is very important in clinical practice since most chemical agents undergo hepatic metabolism. Several potentially toxic agents abound among clinical agents [37] which sometimes make empirical treatment difficult. Also, recent reports have shown both intrinsic and idiosyncratic herb toxicity mechanisms common to traditional and complementary medicines which have been documented on the basis of their predisposing factors [2,3,10]. More so, studies have shown that irrational use of supplement by most patients alone and/or combined with conventional agents are often without the knowledge of the physicians, and may not be reported in the same manner as prescription drugs by patients [35,38,39,7]. Relevant renal function biomarkers including creatinine, urea and uric acid levels did not change in male and female rats administered lowest to highest doses of HEASR used in this study. In the subchronic administrations, creatinine and urea in male and female treated rats were not different from those of control animals. Only the highest dose, HEASR6, produced hypouricaemia in rats of both sexes. An elevated renal biomarker is an indication that poor excretion may occur [40]. This is equally relevant to the hepatic function enzymes since both the liver and kidneys are the major sites for substance elimination in the body. Scientific findings have begun to add to the database on the potential of herbal agents to act as toxicants of interest to different organs, and, in the recent time, some of the traditional and complimentary prescriptions have been contraindicated [10]. In subacute male and female treated rats, HEASR at all doses administered did not alter total protein and albumin levels, however, total bilirubin increased in female but not male rats that received HEASR6. Similarly, serum lipid parameters at both subacute and subchronic administrations did not change in the animals. But, in the female, HEASR5 produced elevated HDL and lowered LDL levels in rats, an indication that HEASR possesses antioxidant potential for lipids metabolism. Treatments with all the doses of the extract both for 28 and 90 days used in this present study boosted haematological parameters in rats. This blood enhancing effects, in part, supports one of its uses in traditional medicine [19]. There was no alteration in body electrolytes in all the treated rats throughout the experiments.

Several medicinal plants have been documented for their reproductive function enhancing properties [41]. Sperm motility was moderately improved and HEASR6 increased sperm counts even when administered for a longer duration than 28 days. However, with HEASR6, there were abnormalities that appeared in the sperm tail in the treated rats. This tendency to improve male sexual life supported our previous findings [20].

Majority of herbal remedies often enjoy longstanding experience testimonies in traditional medicine as evidence of their safety, but unwanted adverse reactions have placed considerable limitations on their use [33]. This is because organ system responsiveness may differ with respect to botanicals contained in them that are so toxic in a large fraction of users [42]. It is more difficult, however, to recognize adverse effects that develop over time in the different systems [33]. Free radicals have a single unpaired electron, highly reactive and as a result of these attract other free radicals or paired electrons readily [24]. This generates a chain reaction of free radicals, leading to damaging biological systems and tissues. More so, HEASR demonstrated pro-oxidation evidenced by an elevated MDA level, a biomarker of oxidative stress, in rats. Studies have shown that reactive oxygen species (ROS) is increased in kidney and systemic circulation on exposure to potentially toxic agents [9,40]. The duration of treatment and/or increasing dosage may cause some level of delayed elimination by the kidney which may explain the increased MDA levels observed in the treated rats, although, the aspect of genetic predisposition cannot be ruled out [9,28,40]. Thus, MDA increased in the kidneys during subacute administration of HEASR5 and HEASR6 treated male rats. HEASR6 also elevated pancreas MDA levels of rats of both sexes. However, when administered in the subchronic treatment, in male rats, MDA increased in HEASR6 in the testis, liver, pancreas, and heart respectively in the male treated rats. Also, the stomach of HEASR5 and HEASR6 treated rats showed elevated MDA levels as well. In the female rats, the subchronic effect of HEASR6 administration caused elevated MDA levels, particularly in the liver and spleen. The level of accumulating ROS in the kidney might surpass other organs in the treated rats. Physiologically, there is a balance between pro-oxidant/oxidant and antioxidant defence systems that enable both cellular and extracellular defence mechanisms respond by inactivating ROS produced in the course of normal conditions [42]. Whereas in pathological conditions, depletion of antioxidant defence system generates ROS including promoting lipid peroxidation, DNA damage, and protein modification which result in tissue damage [43]. Reports that agents that are mainly excreted from kidney may predispose to renal injury have been documented [5,13]. However, the kidney is an organ highly vulnerable to damage caused by ROS, likely due to the abundance of long-chain polyunsaturated fatty acids in the composition of renal lipids [40]. This has generated several attentions in research particularly in understanding the protective antioxidant enzymes and the molecular mechanism of renal diseases [5]. Other conditions which may ultimately result in kidney damage have been discussed extensively in literatures [2]. In the cause of delayed elimination, there are chances that HEASR at one point may serve as an enzyme inhibitor to vital metabolizing enzymes which may be detrimental to life. This is supported by hypouricaemia produced by the highest dose used in this study. Glutathione exists in two forms, as reduced glutathione (GSH) and as oxidized glutathione disulfide (GSSG) [44]. It is believed that the free radicals mopping function is much related to the reduced GSH since it is most abundant in the normal healthy cells and exhibit much greater ratio approaching or greater than a hundred. GSH is a ubiquitous tripeptide composed of glutamate, cysteine, and glycine. It serves to offer anti-toxicity action from exposure to excessive amounts of endogenous and exogenous electrophiles [44]. Besides scavenging free radicals directly, it serves as a cofactor for several other enzymes [43,44]. On the other hand, GSSH is believed to be necessary for providing the appropriate environment for assembly and secretory pathways for proteins [44]. Suggestions are that tissue glutathione levels are often depleted after short-term oxidant exposures but elevated after long-term exposures [43]. This might explain the dose-independent responses observed with HEASR treatment which causes an increase in GSH level in the liver, pancreas, and heart with a concomitant decrease in the lungs. GSH has been shown to participate in other physiological processes including nucleotide metabolism, the formation of lipid second messengers, regulation of nitric oxide homeostasis, and modulation of protein function by redox modification [43,44]. The liver plays a critical role in the metabolism and detoxification of ingested and blood-borne substances [37]. Many drugs, environmental toxicants, and selected dietary components have the potential to cause liver damage by inducing oxidative stress. Though, we now understand that the hepatic stellate cells protect the liver from oxidative stress by synthesizing GSH, but, how tissue injury expands following intoxication has been a long-standing debate [43,45]. In addition, GSH regulates the oxygen equilibrium and the redox status of critical protein sulfhydryl groups for deoxyribonucleic acid repair [43]. Pathophysiological consequences of hepatic oxidative damage include dysregulation of lipid metabolism, impaired liver function and subsequently cell death [13,45]. HEASR demonstrated antioxidant boosting effects seen in some organs where oxidative actions less occurs, particularly in animals that received the lowest dose used in this experiment. In our lab, we recently reported that the antioxidant ability of HEASR, in part, mediated both reproductive behavior and sexual function in male Wistar rats [20]. Plausible as obtained in our present results that HEASR dosing demonstrated systemic toxicity in rats, it may be difficult to conclude, which organs is most involved.

Weight modulation is a very vital characteristic of several medicinal agents [28,30,31]. Here, we explored further the organ weight to body weight ratio. In respect, HEASR6 reduced ovary weight in the subacute female rats. Both HEASR5 and HEASR6 also increased liver and stomach weights respectively. In the subchronic dosing, HEASR6 caused increase in epididymis, pancreas, stomach, and liver weights respectively in the male rats, whereas in the female, HEASR5 and HEASR6 moderately modulated organ weights. More so, HEASR6 further reduced ovary weight in the treated rats.

We previously observed HEASR dosing to improve reproductive indices in male Wistar rats given a 28-day study. Similarly, there was a dose-dependent increase in testosterone levels in rats that received HEASR4, HEASR5, and HEASR6 respectively following subchronic administration in male animals. More so, HEASR6 elevated LH level while FSH level was lowered in all treated rats. On the other hand, in the female rats, the LH, FSH, and oestrogen levels were all improved in a dose-independent manner. In a pragmatic matter, HEASR is a very potent hormonal booster [20].

This present findings evaluated some important biomarkers of liver and renal functions, haematological parameters, electrolytes and endocrine parameters in order to comment on safety. In addition, we estimated cyclooxygenase (COX), a rate-limiting enzyme in the metabolism of arachidonic acid to prostanoids [46]. COX-2 is expressed at the site of inflammation or induced by cytokines, growth factors, and hormones [30,31]. Homeostatic functions and the pathological roles played by COX-2 have been well established [30,46,31]. Like other early-response gene products, it is one of the widest biomarkers used to evaluate the mechanism of toxicity [30,31]. In addition to the baseline parameters, mechanistic toxicity was determined by cyclooxygenase-2 assay; suffice to serve as basis for further research. Following 90 days of administration, both HEASR4 and HEASR5 did not alter COX-2 activity, however, HEASR6 produced an increase in COX-2 activity in treated male and female rats. This provides the suitability that dose-dependent inflammatory action may result from HEASR ingestion. Thus, HEASR may serve as a potential toxicant *in vivo* [30,31].

**Fig. 13:**
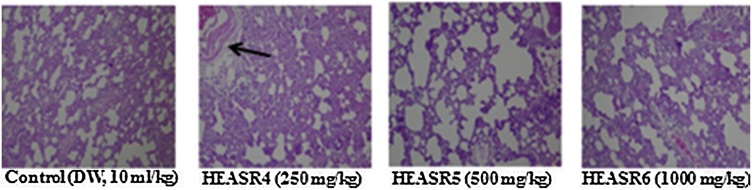
The section of subacute treated male rat lung tissue show air filled alveolar spaces with minimal surrounding interstitial inflammation or congestion of control (distilled water, 10 mL/kg, *p.o.*) (NA); HEASR4 (moderate pulmonary inflammatory); HEASR5 and HEASR6 (mild pulmonary inflammatory). NA: No Abnormality. HEASR = Hydroethanolic extract of *A. smathmannii* root (H & E stain, mag. × 400).

**Fig. 14:**
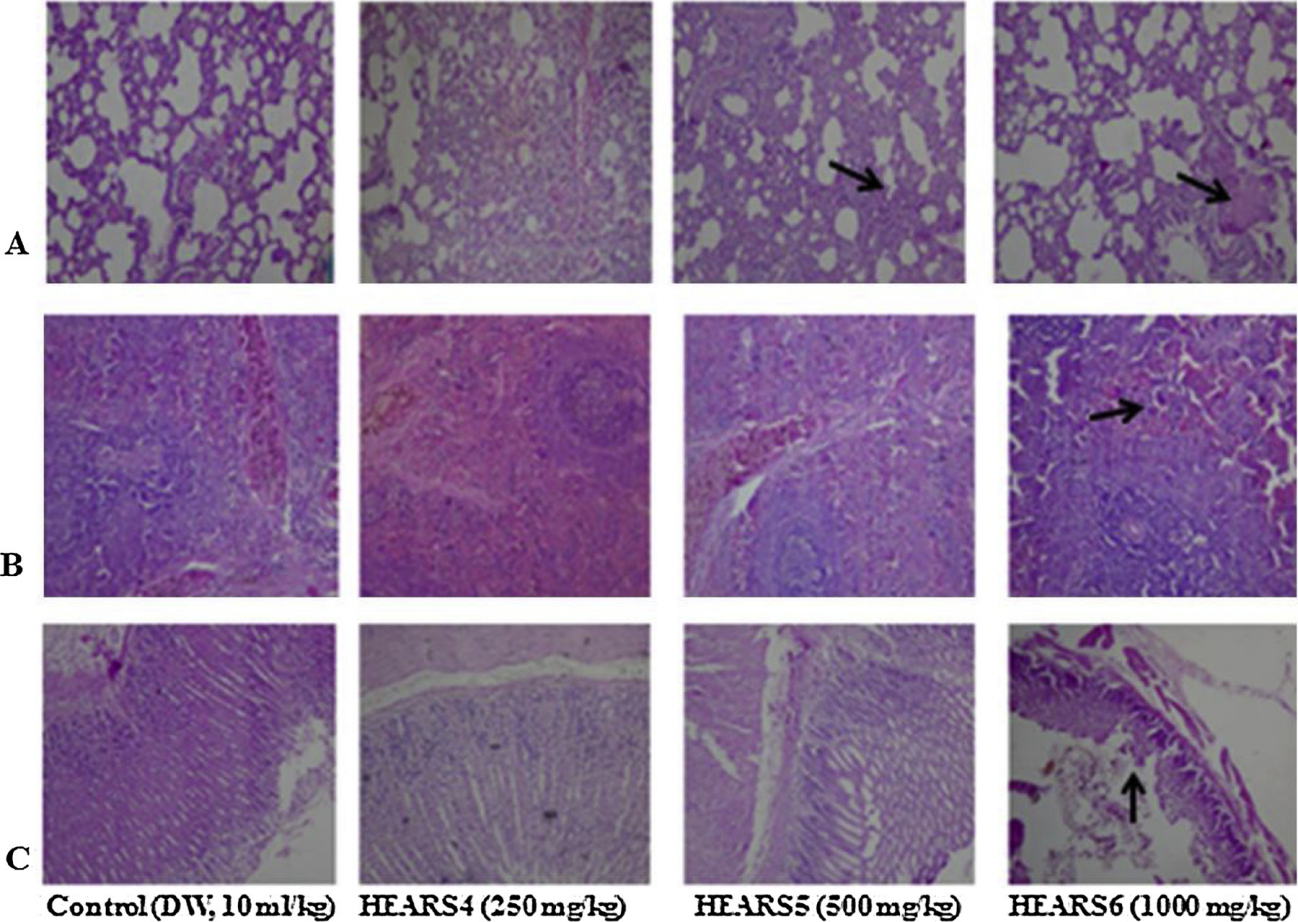
(A) section of subacute treated female rat lung tissue show air filled alveolar spaces with minimal surrounding interstitial inflammation or congestion of (distilled water: DW, 10 mL/kg, *p.o.*) and HEASR4 (NA); HEASR5 and HEASR6 (moderate pulmonary inflammatory) (B) spleen shows lymphoid aggregates which form follicles are seen in control (DW, 10 mL/kg, *p.o.*), HEASR4 and HEASR5 (NA); HEASR6 (areas of surroundin g sinusoidal congestion are seen, Splenic Congestion) (C) section of tissue show mucosal lining and underlying submucosa devoid of inflammatory cell infiltrates with no mucosal ulceration in control (DW, 10 mL/kg, *p.o.*); HEASR4 and HEASR5 (NA); HEASR6 (mucosal erosion). NA: No Abnormality. HEASR = Hydroethanolic extract of *A. smathmannii* root (H & E stain, mag. × 400).

**Fig. 15:**
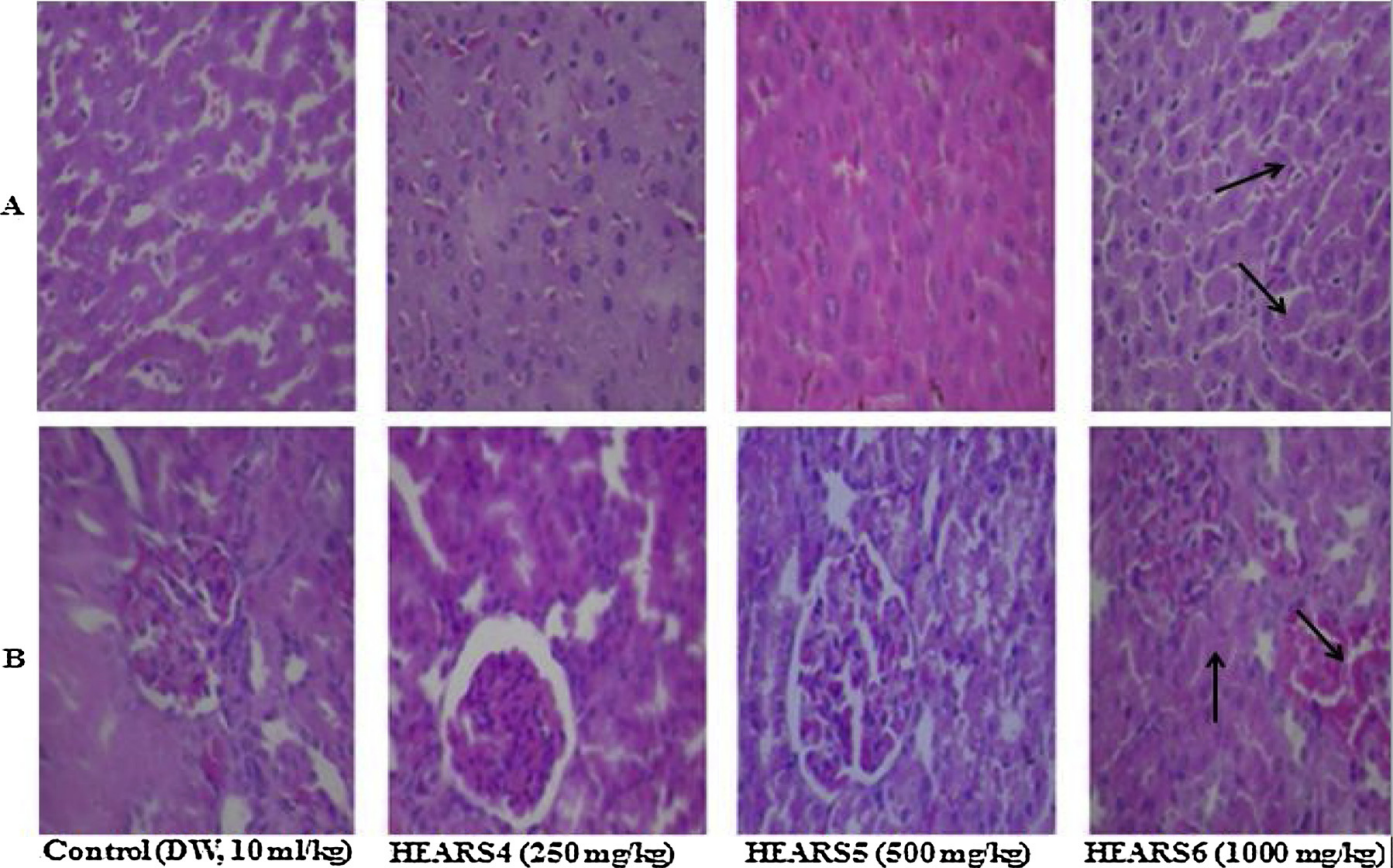
(A) section of subacute treated female rat liver tissue show parallel radially arranged plates of hepatocytes of control (distilled water: DW, 10 mL/kg, *p.o.*), HEASR4 and HEASR5 (NA); HEASR3 (1000 mg/kg) (hepatic sinusoids are packed with red cells, sinusoidal congestion) (B) kidney tissue show normocellular glomerular tufts disposed on a background containing renal tubules of control (DW, 10 mL/kg, *p.o.*), HEASR4, and HEASR5 (NA); HEASR6 (Vascular Congestion). NA: No Abnormality. HEASR = Hydroethanolic extract of *A. smathmannii* root (H & E stain, mag. × 400).

**Fig. 16:**
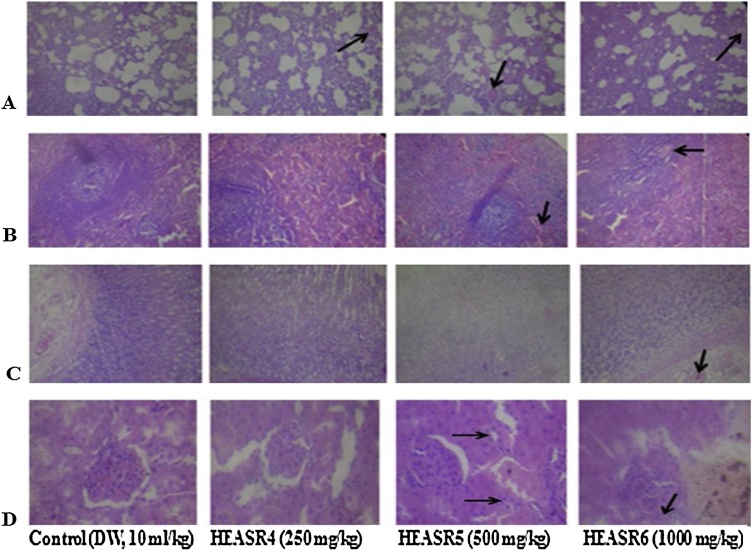
(A) The section of subchronic treated male rats lung tissue show air filled alveolar spaces with minimal surrounding interstitial inflammation or congestion of control (distilled water: DW, 10 mL/kg, *p.o.*) (mild pulmonary inflammatory); HEASR4, HEASR5 and HEASR6 (moderate pulmonary inflammatory) (B) male rats spleen shows lymphoid aggregates which form follicles are seen in control (DW,10 mL/kg, *p.o.*) (NA); HEASR4, HEASR5 and HEASR6 (surrounding sinuses are packed with red cells, splenic Congestion) (C) section of tissue show mucosal lining and underlying submucosa devoid of inflammatory cell infiltrates with no mucosal ulceration in control (DW, 10 mL/kg, *p.o.*), HEASR4 and HEASR5 (500 mg/kg) (NA). HEASR6 shows infiltration of mucosa by dense aggregates of inflammatory cells, as well as congestion of submucosal blood vessels (Gastric Inflammation) (D) section of male rats kidney tissue show normocellular glomerular tufts disposed on a background containing renal tubules of control (DW, 10 mL/kg, *p.o.*) and HEASR4 (NA); HEASR5 (500 mg/kg) and HEASR6 shows congested blood vessels (vascular congestion).

**Fig. 17:**
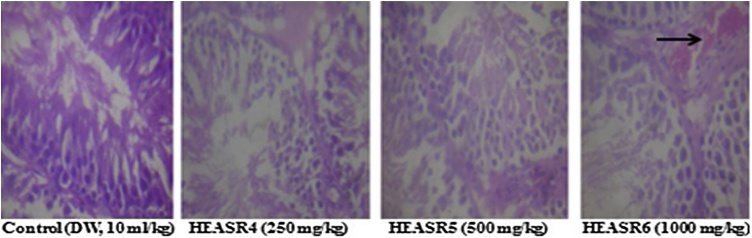
The section of subchronic treated male rats testicular tissue show tubules lined by spermatogenic series cells and containing numerous luminal spermatozoa of control (distilled water, 10 mL/kg, *p.o.*), HEASR4 and HEASR5 (NA); HEASR6 shows interstitium with congested blood vessels, testicular congestion. NA: No Abnormality. HEASR = Hydroethanolic extract of *A. smathmannii* root (H & E stain, mag. × 400).

**Fig. 18:**
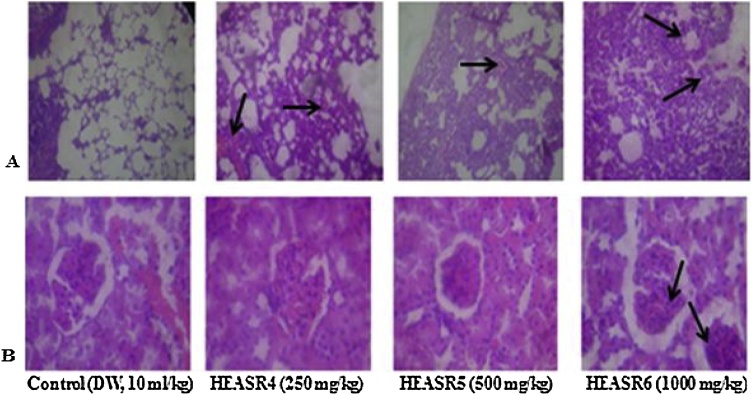
(A) section of subchronic treated female rats lung tissue show air filled alveolar spaces with minimal surrounding interstitial inflammation or congestion of (distilled water, 10 mL/kg, *p.o.*) (NA). HEASR4 shows some reduction in air filled alveolar spaces, with moderate infiltration of interstitium by aggregates of inflammatory cell infiltrates (Mild Pulmonary Inflammation); HEASR5 (Moderate Pulmonary Inflammation); HEASR6 (Severe pulmonary inflammatory) (B) section of female rats kidney tissue show normocellular glomerular tufts disposed on a background containing renal tubules of control (distilled water, 10 mL/kg, *p.o.*), HEASR4 and HEASR5 (NA); HEASR6 shows congested vessels (Vascular Congestion). NA: No Abnormality. E = Hydroethanolic extract of *Acridpcarpus smathmannii* root (H & E stain, mag. × 400).

The rate of mortality forms an important aspect of toxicity study [9]. There was no mortality in subacute dosing for both sexes, however, during a subchronic treatment, mortality occurred in a time and dose-dependent manner. We adduced other death reasons to loss of appetite, oxidative stress, hypodipsia among others. The lowest dose used in this study (250 mg/kg) demonstrated, to a large extent, some level of safety when administered to rodents in a time and dose-dependent manner. This observation may, in part, deduce that the toxicity of HEASR for LOAEL is below 250 mg/kg in various organs in Wistar rats of both sexes. Since we did not examine any reversibility effects of HEASR at the doses administered (250, 500 and 1000 mg/kg) in this present study, it is recommended that future study should assess such possibility. An attempt to evaluate the biochemical and histological effects could be a step towards understanding the interaction of *A. Smeathmannii* with other substances. On the adverse herb reactions, histological assessments show mild to moderate pulmonary inflammation, splenic congestion, mucosal erosion, and vascular congestion respectively following subacute administration. No significant changes in treated liver, kidney, brain, pancreas, spleen, testis, epididymis, stomach and heart of subacutely treated male rats while brain, pancreas, and heart were unaltered in the female rats. In addition, subchronic administration confirmed that HEASR may be associated with lung inflammation, splenic congestion, gastric inflammation, vascular congestion and testicular congestion in rats (Fig. 13, Fig. 14, Fig. 15, Fig. 16, Fig. 17, Fig. 18). No significant changes in subchronic treated liver, brain, pancreas, epididymis, and heart of male rats and in liver, brain, pancreas, spleen, ovary, stomach and heart of female animals. Some of the most abundant bioactive compounds include octadecanoic acid ethyl ester, (*E*)-13-docosenoic acid, octadecanoic acid, 2-hydroxy-1,3-propaned, (E)-9-octadecenoic acid ethyl ester, 9,12-octadecadienoic acid, benzyl alcohol among others [20]. The different possible roles play in the biological system by these agents suggests they should be considered in future studies. Overall, we suggest that study to show how HEASR and/or its bioactive components may interact at the molecular level particularly with specific organs in the body be given attention. This study provides an insight into the possible herb interactions.

## 5 Conclusion

Overall, the LOAEL as obtained in our result is below 250 mg/kg of HEASR for various organs in Wistar rats. Although the toxicity of HEASR is dose and duration dependent, there must be caution with a long duration use of HEASR as evidenced in pneumonitis, gastritis, congestion of liver, spleen, kidney and even testis in this present study.

## Competing interests

Authors declare that they have no competing interests. No specific grant received from any agency in the public, commercial, or not-for-profit sectors.

## Transparency document

Transparency document

## References

[1] World Health Organization (2015). The Selection and Use of Essential Medicines: Report of the WHO Expert Committee, 2015 (including the 19th WHO Model List of Essential Medicines and the 5th WHO Model List of Essential Medicines for Children) (No. 994).

[2] Awodele O., Popoola T.D., Amadi K.C., Coker H.A.B., Akintonwa A. (2013). Traditional medicinal plants in Nigeria—remedies or risks. J. Ethnopharmacol..

[3] Oreagba I.A., Oshikoya K.A., Amachree M. (2011). Herbal medicine use among urban residents in Lagos, Nigeria. BMC Complement. Altern. Med..

[4] Atanasov A.G., Waltenberger B., Pferschy-Wenzig E.M., Linder T., Wawrosch C., Uhrin P. (2015). Discovery and resupply of pharmacologically active plant-derived natural products: a review. Biotechnol. Adv..

[5] Stickel F., Shouval D. (2015). Hepatotoxicity of herbal and dietary supplements: an update. Arch. Toxicol..

[6] Aliakbarzadeh G., Sereshti H., Parastar H. (2016). Pattern recognition analysis of chromatographic fingerprints of Crocus sativus L. secondary metabolites towards source identification and quality control. Anal. Bioanal. Chem..

[7] Buenz E.J., Schnepple D.J., Bauer B.A., Elkin P.L., Riddle J.M., Motley T.J. (2004). Techniques: bioprospecting historical herbal texts by hunting for new leads in old tomes. Trends Pharmacol. Sci..

[8] Akindele A.J., Adeneye A.A., Salau O.S., Sofidiya M.O., Benebo A.S. (2014). Dose and time-dependent sub-chronic toxicity study of hydroethanolic leaf extract of Flabellaria paniculata Cav. (Malpighiaceae) in rodents. Front. Pharmacol..

[9] Kale O.E., Awodele O. (2016). Safety evaluation of Bon-santé cleanser® polyherbal in male Wistar rats. BMC Complement. Altern. Med..

[10] Ekor M. (2014). The growing use of herbal medicines: issues relating to adverse reactions and challenges in monitoring safety. Front. Pharmacol..

[11] Ching C.K., Chen S.P.L., Lee H.H.C., Lam Y.H., Ng S.W., Chen M.L. (2018). Adulteration of proprietary Chinese medicines and health products with undeclared drugs: experience of a tertiary toxicology laboratory in Hong Kong. Br. J. Clin. Pharmacol..

[12] Fugh-Berman A. (2000). Herb-drug interactions. Lancet.

[13] Teschke R., Frenzel C., Schulze J., Eickhoff A. (2012). Spontaneous reports of primarily suspected herbal hepatotoxicity by Pelargonium sidoides: was causality adequately ascertained?. Regul. Toxicol. Pharmacol..

[14] Wang B., Deng J., Gao Y., Zhu L., He R., Xu Y. (2011). The screening toolbox of bioactive substances from natural products: a review. Fitoterapia.

[15] El-Seedi H.R., Burman R., Mansour A., Turki Z., Boulos L., Gullbo J., Göransson U. (2013). The traditional medical uses and cytotoxic activities of sixty-one Egyptian plants: discovery of an active cardiac glycoside from Urginea maritima. J. Ethnopharmacol..

[16] Morton C.V. (1968). A typification of some subfamily, sectional, and subsectional names in the family malpighiacae. Taxon.

[17] Davis C.C., Anderson W.R. (2010). A complete generic phylogeny of Malpighiaceae inferred from nucleotide sequence data and morphology. Am. J. Bot..

[18] Van Andel T.R., Croft S., Van Loon E.E., Quiroz D., Towns A.M., Raes N. (2015). Prioritizing West African medicinal plants for conservation and sustainable extraction studies based on market surveys and species distribution models. Biol. Conserv..

[19] Catarino L., Havik P.J., Romeiras M.M. (2016). Medicinal plants of Guinea-Bissau: therapeutic applications, ethnic diversity and knowledge transfer. J. Ethnopharmacol..

[20] Kale O.E., Awodele O., Akindele A.J. (2019). *Acridocarpus smeathmannii* (DC.) Guill. & Perr. root enhanced reproductive behaviour and sexual function in male Wistar rats: biochemical and pharmacological mechanisms. J. Ethnopharmacol..

[21] Kilkenny C., Browne W., Cuthill I.C., Emerson M., Altman D.G. (2011). Animal research: reporting in vivo experiments—the ARRIVE guidelines. J. Cereb. Blood Flow Metab..

[22] Beutler E., Duron O., Kelly B.M. (1963). Improved method for the determination of blood glutathione. J Lab Clin Med.

[23] Varshney R., Kale R.K. (1990). Effect of calmodulin antagonist on radiation induced lipid peroxidation in microsomes. Int. J. Radiat. Biol..

[24] Scoditti E., Nestola A., Massaro M., Calabriso N., Storelli C., De Caterina R., Carluccio M.A. (2014). Hydroxytyrosol suppresses MMP-9 and COX-2 activity and expression in activated human monocytes via PKCα and PKCβ1 inhibition. Atherosclerosis.

[25] De Smet Peter A.G.M. (2004). Health risks of herbal remedies: an update. Clin. Pharmacol. Ther..

[26] Skalicka-Woźniak K., Georgiev M.I., Orhan I.E. (2017). Adulteration of herbal sexual enhancers and slimmers: the wish for better sexual well-being and perfect body can be risky. Food Chem. Toxicol..

[27] Shaw D. (2010). Toxicological risks of Chinese herbs. Planta Medica.

[28] Akindele A.J., Unachukwu E.G., Osiagwu D.D. (2015). 90 Days toxicological assessment of hydroethanolic leaf extract of Ipomoea asarifolia (Desr.) Roem. and Schult.(Convolvulaceae) in rats. J. Ethnopharmacol..

[29] Awodele O., Badru W.A., Busari A.A., Kale O.E., Ajayi T.B., Udeh R.O., Emeka P.M. (2018). Toxicological evaluation of therapeutic and supra-therapeutic doses of Cellgevity® on reproductive function and biochemical indices in Wistar rats. BMC Pharmacol. Toxicol..

[30] Kale O.E., Akinpelu O.B., Bakare A.A., Yusuf F.O., Gomba R., Araka D.C. (2018). Five traditional Nigerian Polyherbal remedies protect against high fructose fed, Streptozotocin-induced type 2 diabetes in male Wistar rats. BMC Complement. Altern. Med..

[31] Kale O.E., Oyesola T.O., Raji F.S. (2018). Celecoxib, a cyclooxygenase-2 inhibitor, offers chemoprevention against reproductive and neurobehavioural abnormalities induced by atrazine in male Wistar rats. Environ. Toxicol. Pharmacol..

[32] Krewski D., Acosta D., Andersen M., Anderson H., Bailar J.C., Boekelheide K. (2010). Toxicity testing in the 21st century: a vision and a strategy. J. Toxicol. Environ. Health Part B.

[33] Jordan S.A., Cunningham D.G., Marles R.J. (2010). Assessment of herbal medicinal products: challenges, and opportunities to increase the knowledge base for safety assessment. Toxicol. Appl. Pharmacol..

[34] Lu F.C., Jessup D.C., Lavallee A. (1965). Toxicity of pesticides in young versus adult rats. Food Cosmet. Toxicol..

[35] Awodele O., Agbaje E.O., Abiola O.O., Awodele D.F., Dolapo D.C. (2012). Doctors’ attitudes towards the use of herbal medicine in Lagos, Nigeria. J. Herb. Med..

[36] Awodele O., Oreagba I.A., Odoma S., da Silva J.A.T., Osunkalu V.O. (2012). Toxicological evaluation of the aqueous leaf extract of Moringa oleifera Lam. (Moringaceae). J. Ethnopharmacol..

[37] Larrey D. (2000). Drug-induced liver diseases. J. Hepatol..

[38] Gurib-Fakim A. (2006). Medicinal plants: traditions of yesterday and drugs of tomorrow. Mol. Asp. Med..

[39] Shelley B.M., Sussman A.L., Williams R.L., Segal A.R., Crabtree B.F. (2009). ’They don’t ask me so I don’t tell them’: patient-clinician communication about traditional, complementary, and alternative medicine. Ann. Fam. Med..

[40] Shimoishi K., Anraku M., Kitamura K., Tasaki Y., Taguchi K., Hashimoto M. (2007). An oral adsorbent, AST-120 protects against the progression of oxidative stress by reducing the accumulation of indoxyl sulfate in the systemic circulation in renal failure. Pharm. Res..

[41] Bella A.J., Shamloul R. (2014). Traditional plant aphrodisiacs and male sexual dysfunction. Phytother. Res..

[42] Abdel-Rahman A., Anyangwe N., Carlacci L., Casper S., Danam R.P., Enongene E. (2011). The safety and regulation of natural products used as foods and food ingredients. Toxicol. Sci..

[43] Mehendale H.M. (2012). Once initiated, how does toxic tissue injury expand?. Trends Pharmacol. Sci..

[44] Pompella A., Visvikis A., Paolicchi A., De Tata V., Casini A.F. (2003). The changing faces of glutathione, a cellular protagonist. Biochem. Pharmacol..

[45] Ekor M., Odewabi A.O., Kale O.E., Oritogun K.S., Adesanoye O.A., Bamidele T.O. (2011). Pharmacologic inhibition of the renin-angiotensin system did not attenuate hepatic toxicity induced by carbon tetrachloride in rats. Hum. Exp. Toxicol..

[46] Capra V., Rovati G.E., Mangano P., Buccellati C., Murphy R.C., Sala A. (2015). Transcellular biosynthesis of eicosanoid lipid mediators. Biochim. et Biophys. Acta (BBA)-Mol. Cell Biol. Lipids.

